# Intrapreneurship and technological innovation in optimizing qualitative research as evidenced at Infectious Diseases Institute, Uganda

**DOI:** 10.1186/s13731-021-00188-y

**Published:** 2021-12-05

**Authors:** Kenneth Mulungu, Proscovia Katumba, Rosalind Parkes Ratanshi, Adelline Twimukye, Barbara Castelnuovo, Aidah Nanvuma, Godfrey Akileng

**Affiliations:** 1 Infectious Diseases Institute, Kampala, Uganda; 2 Makerere University, Kampala, Uganda

**Keywords:** Intrapreneurship, Technological innovation, Qualitative research optimization, Transcription and translation errors, Business process reengineering

## Abstract

**Background:**

Discrepancies between what is transcribed and the actual interview recordings were noticed in qualitative research reports. This study aimed at the development of a new transcription software (Jiegnote), and the evaluation of its effectiveness in the optimization of the transcription process, to minimize transcription completion time, and errors in qualitative research.

**Methods:**

The study was a mixed methods project implemented from September to November 2020. The qualitative aspect of the study was phenomenological in perspective whereas the quantitative consisted of a randomized controlled trial (RCT) with a parallel design.

**Results:**

At the time of the study, the Jiegnote software was a working prototype. We enrolled a total of 26 participants; 14 participants had their data analyzed in the RCT part of the study, 13 participated in the in-depth interviews, and 22 in the answering of Semi Structured Questionnaires. Upon the execution of an independent *t* test, results showed that, there was no statistical significance between the intervention and control means. On considering the total average transcription completion time and the type of language in which an audio case was recorded, the effect size evaluation implied that the Jiegnote software had a small impact (Hedges' *g* = 0.413438) in reducing the total average time taken to translate and transcribe audio cases that were recorded in a local language (Luganda), and a large impact (Hedges' *g* = 1.190919) in reducing the total average time taken to transcribe audio cases that were recorded in a foreign language (English). On considering the total average number of transcription errors and the type of language in which an audio case is recorded, the effect size evaluation implied that the Jiegnote software had a small impact (Hedges' *g* = 0.213258) in reducing the total average time taken to translate and transcribe audio cases that were recorded in a local language (Luganda). This was further observed (Hedges' *g* = 0.039928) in the transcription of cases that were recorded in a foreign language (English). On considering the in-depth interview data outcomes, participants responded that the Jiegnote software media looping functions (algorithm) enabled them to accomplish their transcription tasks in a shorter time and with fewer errors compared to the traditional methods.

**Conclusion:**

The study demonstrates utilities associated with intrapreneurship and technological innovation in an organization setting whereby, the Jiegnote technology that was developed by the researchers, had some impact on the optimization of the qualitative research value chain. This was observed through the effect size (impact) evaluations that were conducted to investigate the superiority of the Jiegnote software against the traditional transcription methods, in minimizing the average number of errors committed, and time taken to complete a transcription process.

## Background

Transcription involves processes that convert audio data into text, and achieving quality utility outcomes in transcription requires skilled labor.

Constraints associated with achieving quality outcomes from qualitative research processes have been observed whereby, discrepancies between what is transcribed and the actual audio interview recording, exist in some qualitative research study initiatives (Holstein & Gubrium, 2003).

Studies implicate that, some researchers hardly give transcription quality, a priority (Holstein & Gubrium, 2003) and that, transcriptionists mistake some wordings for others that are not in line with the interview context due to the poor recording quality. Furthermore, the process of running recorded information on audio players; back and forth, to replay a section of an audio file during transcription, leads to omission and the misinterpretation of some words. It was further observed that, the placement of a coma in a phrase sometimes altered the meaning of a sentence. Transcripts never exhibited expressions like; intonation, mimicking of people, or respondents quoting others, therefore, annotations were omitted in some transcribed data. (Holstein & Gubrium, 2003).

Both naturalism and denaturalism make up the two domains of the transcription process. (Oliver et al., 2005). With naturalism, all utterances are captured in the text while with denaturalism, idiosyncratic elements of speech like pauses and nonverbal sentiments are omitted. On choosing between the two domains, researchers argued that the research question influenced one’s preference and that denaturalism leads to the omission of information that could be relevant in data analysis.

There are difficulties in; decoding pronunciation, vocalizations, non-verbal communication, and irregular grammar during the transcription process (Oliver et al., 2005). With pronunciation, slangs are used, and upon the recording process, the audio recorders poorly store the data. Furthermore, geo-ethnic accents create misunderstanding and confusion during the transcription of conversations.

The transcription process is time and labor intensive. Generally, this process is implemented by a variety of professionals like; administrative staff, graduate students, and non-research professionals with varying skills, training, and supervision; hence, posing the risk of poorly transcribed data (Hennink & Weber, 2013). Furthermore, the use of multiple transcribers led to the variance in quality, and content of the transcribed data.

Uganda is a multilingual and a multicultural country, with more than thirty spoken native languages. The languages can be grouped into three main categories; Bantu, Central Sudanic, and Nilotic. (Sawe, 2017). The multilingual nature of Uganda poses a risk of improper translation and transcription of data during qualitative research service provision.

The Infectious Diseases Institute (IDI), a non-government organization (NGO), in Kampala, Uganda, is a healthcare research setting that conducts; quantitative, qualitative and mixed methods research projects in various African communities.

The traditional transcription process at IDI, requires employees to work with two computer programs; a word processor (Microsoft Word), and an audio player (Microsoft Windows Media Player). Researchers and their assistants switch back and forth between the word processor and the audio player to convert audio data into text (transcription), consequently, this makes the transcriptionists less productive due to the iteration between the audio player and the word processor applications. This is evidenced with the longer time frames to accomplish the transcription processes in some research projects. The iterative process between two computer applications in the traditional transcription methods and the operational gap of longer transcription time, is due to the lack of a single dedicated optimization software to aid employees in the easier, and efficient implementation of transcription tasks during qualitative research activities. As staff and researchers at IDI, we decided to solve the transcription bottleneck through intrapreneurship and technological innovation.

With the assumption that an intrapreneural organizational work approach enhances labor productivity and innovation, the study aimed at the development of a new transcription software called Jiegnote, and the evaluation of its effectiveness in the transcription of audio files, at the Infectious Diseases Institute, in Kampala, Uganda.

## Literature review

### Intrapreneurship and innovation

Intrapreneurship refers to initiatives by employees in organizations to undertake new business activities (Bosma et al., 2010). These employees are inventive and are entrepreneurial within organization perimeters (Desouza, 2011).

Intrapreneurship is a subsection of the corporate entrepreneurship concept, unfortunately, there is little literature focused on the provision of guidance to employees and managers on how to implement it, and that, few managers are able to articulate and communicate the process of intrapreneurship to their subordinates (Desouza, 2011). Some studies implicate that intrapreneural organizations endeavored to provide resources (Finance and time) to their employees to work on new ideas that could be valuable to an organization’s development, however, such support was not observed in most of other firms that were not intrapreneural (Desouza, 2011).

Intrapreneurship is a multilevel construct that affects an organization at any level of its structure and that, it is inclined towards the bottom up management approach. Many research studies on intrapreneurship are limited for their focus is highly directed towards an organization’s characteristics, hence excluding factors associated with individual (employee) characteristics (Neessen et al., 2019).

A study on entrepreneurship by Bosma (2010) and his team implicated that intrapreneurship was prominent in high income countries as compared to low income ones, and that, intrapreneurs possessed entrepreneurial perceptions and attitudes, as a result, low income countries miss benefits associated with intrapreneurship. The study further stipulated that intrapreneurship was a special type of entrepreneurship with a number of shared behavioral characteristics, however, intrapreneurship faced the limitation of corporate hierarchy as it belonged to the domain of employee behavior.

Intrapreneurship and innovation disciplines complement each other for they all involve creation of products or services. There are a lot of arguments (discrepancies) pertaining to the right definition of an innovative product or service, and therefore, innovation definitions have evolved depending on the stage of development of industries. In the early 1960s, the innovation discipline was defined in line with the manufacturing industry whereby research and development led to new products that were patented, and that, an innovation was generally acknowledged in terms of a physical product that was new (Schachter, 2018).

Unlike the 1960s views on innovation, scholars in the modern age of industrial development, view innovations in multiple contexts. Some scholars have argued that for something to be innovative, it should include some or all of the following factors; novelty, invention, or change (Schachter, 2018), and that an innovation can either be tangible or intangible.

In their published book, Ton et al. (2005), described innovation as an idea, practice, or object that is perceived as new by an individual or other unit of adoption, and that, the characteristics of an innovation, as perceived by the members of a social system, determines its rate of adoption.

According Weisberg (2006), for something to be an innovative (creative) product, it must be new and that, there are two novelties that must be separated in the creative process, that is to say; novelty of a person, versus that of the world. If an individual developed a product and he or she was not aware that the product had been developed somewhere else, then that individual is creative for the idea of the product was novel to him or her, therefore, Weisberg argues that for a product to be creative or innovative, it should encompass novelty by the creator, and that a creative person is one who produces such products. Furthermore, Weisberg stipulated that if a product was created accidentally without intentional novelty, then the incidence is not creativity.

From the literature review, we observe that both, intrapreneurship, and innovation involve artifact development, and that, the definition of an innovation depends on the development context of the product or service of the creator (innovator) in a social system, however, scholars conclude that at least, an innovative item has to be novel. In this study, we define an innovation as something that is new or improved in such a way that the target population for the innovative item acknowledges the novelty of the new product or service. Furthermore, we define intrapreneurs as people who voluntarily come up with new developmental concepts (ideas, services or products) within their host organizations regardless of their position (manger, or not a manager) and the type of organization (profit, or not for profit).

## Theoretical framework

This study involved the development of a new product (Jiegnote) and the evaluation of its effectives in the optimization of the qualitative research value chain through reduced transcription errors and completion time compared to the traditional transcription methods. Therefore, the new product development theory was used as a theoretical framework for the research project.

### New product development theory

According to Gurbuz (2018), a product is an object or service which is functional and emotional to satisfy the customer’s need, avail value, and be delivered as the way customer demands. Products take shape, and hence they can either be tangible or intangible. New product development can result into a new profit or a loss however, it was noted that if a new product was introduced at the right time in the market, it was more likely to create significant amounts of profits to the host organization (Gurbuz, 2018).

Gurbuz further stipulated that a new product can take several categorizations which may include; (1) Major innovations, (2) Product improvements, (3) Product additions, and (4) Repositioned products, and that, the new product development process undergoes eight stages, and in each stage, business decisions should be critically made to guide the next step in the implementation of another stage.

#### Stage 1: Generation of new product ideas

This stage involves coming up with a series of new product ideas. Ideas can come from; research and development departments, competitors, customers and employees among other sources. The Jiegnote software idea was generated through the observation of the transcription processes at IDI. The Idea was then forwarded to the research department management for analysis.

#### Stage 2: Screening and evaluation of ideas

In this stage, ideas are screened and priority is given to those that meet the higher value depending on the organization’s priority matrix. The IDI research department evaluated the Jiegnote software idea within 14 days from the time of its initial submission.

#### Stage 3: Concept development and testing

In this stage, the screened and selected ideas are organized in a detailed and meaningful form. Potential customers’ thoughts are taken up pertaining to the new product initiative, and the product concept that receives the best score, is selected as the new product to be developed. Detailed documentation of the Jiegnote transcription software idea was made, and a group of qualitative researchers were selected to provide information pertaining to the usefulness of the software in their work processes. Upon the validation of the Jiegnote value proposition, a decision was made to have the sample (working prototype) version of the software developed.

#### Stage 4: Marketing strategy

This stage involves establishing a marketing strategy for the considered concept. A series of factors are put into consideration. These may include; profitability, price, distribution and market for the new product. Investigations associated with factors like; the propensity to buy, to recommend, and to adopt the Jiegnote software, were conducted by issuing self-administered questionnaires to potential clients (Researchers and their assistants).

#### Stage 5: Business strategy/analysis

In this stage, projections on sales, profits and costs, pertaining to the new product development process are made. If the analytics indicate significant (positive) returns in line with the business objectives, then stage 6 on product development is initiated. The Data from the questionnaires pertaining to the Jiegnote software value proposition was analyzed, and the outcomes indicated that, there was a higher propensity to consume the software.

#### Stage 6: Product development

A Sample of the new product is created and tested amongst customers to evaluate its attractiveness, and potential uptake in the market. The Jiegnote software was developed to a minimal viable product (MVP) level through agile software development process. (Akbar et al., 2018).

#### Stage 7: Test marketing

Tests are made in this stage to guide the appropriate and effective marketing initiatives that will maximize returns on investment. This stage helps to minimize unnecessary costs that may impede high profitability in the commercialization stage. This study was conducted among 26 IDI transcribers. The study data was to be used in the development of an effective strategy that would achieve a high consumption (adoption) rate of the Jiegnote software in the future.

#### Stage 8: Commercialization

This stage involves determining when to introduce the new product into the market. Considerations are made pertaining to the scale of introduction of the product to the market. It may be regional, national or international scale.

**Fig. 1:**
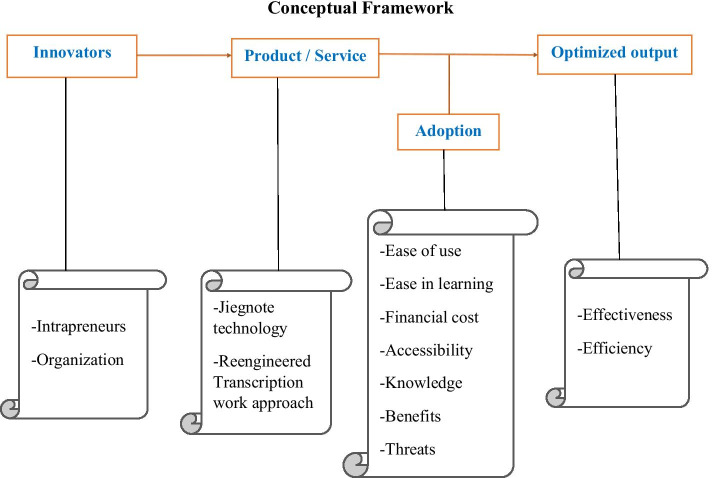
Jiegnote conceptual framework

The framework (Fig. 1) lists three factors; innovators, products/services, and adoption, that influence a desired outcome of optimized transcription process in qualitative research.

*Innovators* Intrapreneurs work within their employers’ boundaries and hence, the organization’s environment plays a significant role in influencing what is to be innovated. This is why both the intrapreneurs and the organization are categorized under innovators in the framework.

*Product/service* The new products and services are the innovations created by innovators. In this study, the Jiegnote software is the new product that creates a new transcription work approach (service) in qualitative research value chain of IDI, Uganda.

*Adoption* This section represents the perceptions of the potential adopters for the new products and services. The perceptions include; ease of use, ease in learning, financial cost, accessibility, knowledge, benefits and threats among others.

*Optimized output* The optimized output is the target desired outcome due to the three preceding factors as listed in Fig. 1. The effectiveness and efficiency are the broad factors used to evaluate the impact of the predictor factors. Time taken to transcribe audio data using the Jiegnote software compared to the traditional methods falls under the efficiency factor and the proportions of transcription errors observed when using the Jiegnote software compared to the traditional methods falls under the effectiveness factor.

## Methodology

We used mixed methods in the research project. This was aimed at assessing whether we could get related outcomes in the qualitative and experimental parts of the study (Regnault et al., 2018; Schoonenboom & Johnson, 2017). Furthermore, we wanted to investigate the study topic in multiple contexts (O’Cathain et al., 2007). The qualitative aspect of the study was phenomenological in perspective for the lived experiences of participants in line with the Jiegnote software were evaluated whereas the quantitative component of the study consisted of a randomized controlled trial (RCT) with a parallel design (Spieth et al., 2016). Furthermore, the RCT was used to measure the effectiveness of the Jiegnote software intervention in a transcription process (Hariton & Locascio, 2018).

The study was conducted from September to November 2020 at the Infectious Diseases Institute (IDI), in Kampala, Uganda, which is part of Makerere University, College of Health Sciences. The organization hosts qualitative, quantitative and mixed methods researchers who were important in giving responses during study activities.

Our sample population consisted of IDI staff in the behavioral science peer group. The peer group involves trained professionals responsible for the transcription and translation of audio files in some IDI research projects.

To meet the study inclusion criteria, participants had to have prior experience in the transcription of audio recordings, provide a written informed consent, and had to be aged 18 years and above. Participants who did not comply to study procedures were excluded from the study.

## Study procedures and data collection

### The Jiegnote Software development

The Jiegnote software was expected to read videos and audios, and furthermore, record texts concurrently to allow transcriptionists transcribe data with only one computer program. The software was also expected to effect a media looping algorithm that facilitates audio clarity, and the convenient creation and working within audio loops, during the transcription process, hence preventing transcribers from switching back and forth, between a word processor, and an audio player when converting audio information into text. Therefore, Jiegnote, a single automated software application, was intended to provide; an efficient work process, minimal errors, and reduced time in the transcription process, compared to the traditional transcription methods that were labor intensive, error prone, and required the use of two software applications when transcribing audio data.

In relation to the new product development theory (Gurbuz, 2018), the data collection tools were pretested and piloted whereby, a group of 5 participants who had prior experience in transcription was formed, and later interviewed in line with the observed transcription challenges. The generated data helped us to understand the inefficiencies of the traditional transcription methods, and furthermore, we were able to develop the Jiegnote transcription software at a minimal viable product level.

The software incorporated an audio player and a word processor on one interface, therefore, the end-users worked with only one tool when transcribing their recorded narratives, unlike the traditional methods that involved clients working with two separate software applications. Furthermore, the Jiegnote looping algorithm was developed in a way that ensured information reemphasis and clarity, to minimize transcription omission and insertion errors.

### Jiegnote software evaluation

Study participants were randomized into two groups. Four audio cases which are; case a, b, c and d, were used for transcription by the groups. The first two cases were in Luganda language and the remaining in English.

Participants were assigned study Identification numbers (IDs) that were confidential. The investigator and research assistants were not able to tell participant names that corresponded to the study identification numbers. The participants were informed about the confidentiality. This was intended to mitigate any potential bias or confounding factors during the answering of questions since the lead researcher was a co-worker to the study respondents.

The Jiegnote software was presented to the study participants who were trained on how to use it in the transcription process. A return to demonstration was effected among participants when learning the software. The audio files in Luganda language were translated and transcribed by participants to English during the study activities.

Fast pacing is when an audio file speed is altered to an extent that it is faster than its original recorded state. For example, an audio file playing at 25 s per frame can be fast paced to 50 s per frame. “A” and “C” cases were fast paced whereby, their original speeds were doubled using an audio file editor, unlike “B” and “D”.

The lead researcher and an experienced senior qualitative researcher transcribed all the four audio cases to create four written transcripts (master files), that were later used as benchmarks in determining the differences in outcomes (transcription errors and completion time), between the use of the Jiegnote software and the traditional transcription methods among respondents.

Participants were randomly categorized into two groups that were given the four audio case files; a, b, c and d, to transcribe using the Jiegnote software and the traditional methods. There was timing of sessions to determine duration taken to finish the transcription of each audio case per participant. The study ensured that each participant transcribes two audio cases using Jiegnote software (intervention) and two other ones using the traditional method (control).

In the last stage of the Jiegnote software evaluation, questionnaires were issued, and in-depth interviews conducted among participants. This stage’s inclusion criterion stipulated that; a participant should have transcribed at least one audio case using the Jiegnote software.

## Data analysis

When considering the quantitative component of the study, we used an independent t test to evaluate the statistical significance between the intervention and control group averages (Kim, 2015). The Hedge's *g* test was used to determine the magnitude of the intervention on the total number of transcription errors and completion time (Brydges, 2019).

On considering the qualitative component of the study; transcribed content from the use of the software versus the traditional method was evaluated for accuracy (errors) and time of completion. The transcription errors were categorized into word and annotation errors. These categories were further broken down into omission and insertion errors. Word omission error is when a transcriber misses out a word or series of words to an extent that meaning of the original recorded narrative is altered. Annotation omission error is when a transcriber misses out an annotation or series of annotations to an extent that meaning of the original recorded narrative is altered. Word insertion error is when a transcriber adds a word or series of words into a transcript to an extent that the meaning of the original recorded narrative is altered. Annotation insertion error is when a transcriber adds an annotation or series of annotations into a transcript to an extent that meaning of the original recorded narrative is altered.

During the In-depth interview data analysis study stage, the transcribed recorded audio data and the text one from self-administered questionnaires were analyzed using the inductive thematic analysis method. Transcript texts of interest were varyingly highlighted by coloring, and codes generated with brief description of the highlighted phrases. The created codes were evaluated to identify patterns and hence facilitate the development of themes, however, some codes were grouped under a single theme depending on their patterns.

The themes were later reviewed to assess their usefulness to the study and a final list of them was created, named and defined to bring out meaning. This data analysis stage of the study involved three researchers that independently generated study codes and themes per transcript, to limit potential bias when yielding meaning from the qualitative data. The final themes had to be agreed upon by all the three researchers to be part of the study results.

## Ethical considerations

### Institutional Review Board

The study was approved by the School of Biomedical Science, REC (SBS-783) and the Uganda National Council for Science and Technology (SIR38ES).

## Results

### Study enrolment

We enrolled 26 participants; 13 participated in the in-depth interviews and 22 in the answering of Semi Structured Questionnaires. Out of the 14 participants in the randomized controlled trial; group one consisted of 8 while group two consisted of 6 participants.

### Transcription errors, and completion time

Tables 1 and 2, show the time taken by each participant to complete the transcription of the audio cases; A, B, C and D; and Tables 3 and 4 show the total number of errors committed when using the Jiegnote software, and the traditional methods.

**Table 1:** The translation and transcription completion time for cases; A, and B

Participants (IDs)		Traditional methods (control)	Jiegnote (intervention)
		Completion time (min)	
Group 1	Group 2	Group 1	Group 2
01	13	176	183
02	06	129	191
07	15	160	211
10	18	193	72
11	25	202	141
17	20	130	140
23		143	
26		287	
Total		1420	938
Average		177.5	156.3

**Table 2:** The Transcription completion time for cases; C, and D

Participants (IDs)		Jiegnote (intervention)	Traditional methods (control)
		Completion time (min)	
Group 1	Group 2	Group 1	Group 2
01	13	141	283
02	06	157	315
07	15	137	220
10	18	138	112
11	25	124	157
17	20	101	131
23		113	
26		173	
Total		1084	1218
Average		135.5	203

**Table 3:** Transcription errors committed during cases; A, and B

Participants (IDs)		Traditional methods (control)	Jiegnote (intervention)
		Number of errors	
Group 1	Group 2	Group 1	Group 2
01	13	161	152
02	06	230	162
07	15	180	208
10	18	208	227
11	25	164	207
17	20	243	214
23		222	
26		204	
Total		1612	1170
Average		201.5	195

**Table 4:** Transcription errors committed during cases; C, and D

Participants (IDs)		Jiegnote (intervention)	Traditional methods (control)
Group 1	Group 2	Group 1	Group 2
01	13	106	94
02	06	126	107
07	15	72	130
10	18	129	157
11	25	118	143
17	20	131	140
23		189	
26		167	
Total		1038	771
Average		129.75	128.5

For audio cases; A, and B, translation was required, for they were recorded in a local language. Each participant worked with the Jiegnote software (intervention), and the traditional methods (control), at different time points. The original speeds for cases; C, and A, were doubled before the intervention and control study stages.

### Transcription completion time

When considering the arithmetic average taken to translate and transcribe cases; A, and B, group 2 that used the Jiegnote software (intervention) finished in a shorter time (156.3 min) compared to group 1 that used the traditional method (177.5 min), (*p* = 0.46).

Upon the execution of an independent t test, results showed that, there is no statistical significance between the intervention and control means, and therefore the data outcomes were not sufficient to affirm the superiority of the Jiegnote software.

Upon the evaluation of the effect size, results indicate that the Jiegnote software had a small effect (Hedges' *g* = 0.413438) in reducing the total average time taken to translate and transcribe the audio cases that were recorded in a local language (Luganda).

When considering the arithmetic average time taken to transcribe cases; C, and D, group 1 that used the Jiegnote software (intervention) finished in a shorter time (135.5 min) compared to group 2 that used the traditional method (203 min), (*p* = 0.11). Furthermore, the effect size outcomes indicate that the Jiegnote software had a large effect (Hedges' *g* = 1.190919) in reducing the total average time taken to transcribe audio cases that were recorded in a foreign language (English).

When considering average completion time, whenever a group got assigned with the Jiegnote technology, it performed better than its counterpart using the traditional methods by having lesser total time taken to complete a transcription process, regardless of the language used in the recorded audio cases (Tables 1 and 2).

Upon the execution of the independent t tests, results showed that, regardless of the language used in a recorded audio narrative, there were no statistical significances between the intervention and control means, and therefore, the data outcomes were not sufficient enough to affirm the superiority of the Jiegnote software in reducing the transcription completion time.

### Transcription errors

When considering the total average number of errors committed during the translation and transcription of cases; A, and B, group 2 that used the Jiegnote software (intervention) committed fewer transcription errors compared to group 1 that used the traditional method (*p* = 0.70). Upon the execution of an independent t test, results showed that, there is no statistical significance between the intervention and control means, and therefore the data outcomes were not sufficient to affirm the superiority of the Jiegnote software.

Upon the evaluation of the effect size, results indicate that the Jiegnote software had a small effect (Hedges' *g* = 0.213258) in reducing the total average time taken to translate and transcribe the audio cases that were recorded in a local language (Luganda).

When considering the total average number of errors committed during the transcription of cases; C, and D, group 2 that used the traditional methods (control) committed fewer transcription errors (128.5) compared to group 1 that used the Jiegnote software (129.75), (*p* = 0.94).

Upon the execution of the independent t tests, results showed that there were no statistical significances in the total average numbers of errors committed between the control and intervention sample populations, and therefore, the data outcomes were not sufficient to affirm the superiority of the Jiegnote software in reducing the transcription errors.

Upon the evaluation of the effect size, results indicate that the Jiegnote software had a small effect (Hedges' *g* = 0.039928) in reducing the total average number of errors committed during the transcription of cases that were recorded in a foreign language (English).

### Speed versus errors (quality)

All participants who took the minimum time to complete the transcription of their respective audio cases committed more total transcription errors, and so yielded poorer quality of transcripts compared to their counterparts who took the maximum transcription time on similar cases regardless of the transcription method being used (Table 5). In group 1, participant 02 on case “A” took the least transcription time but committed more errors (101 transcription errors) compared to participant 26 in the same group on case “A” who took the maximum transcription time but committed less errors (95 transcription errors). Considering Table 5, there was an inverse relationship between speed and quality of transcript per audio case completed by a transcriber.

**Table 5:** The relationship between transcription time (maximum and minimum time) and total number of errors committed by participants

Group 1					Group 2			
Cases	Errors	(Time of completion)		Errors	Errors	(Time of completion)		Errors
		Maximum time	Minimum time			Maximum time	Minimum time	
		Participants				Participants		
A	95	26	02	101	77	06	18	106
B	109	26	17	118	105	15	18	121
C	74	26	17	83	73	06	20	87
D	25	01	23	106	33	13	18	61

We asked participants on how effective they found the software compared to the traditional methods. The following themes represent their narratives (subjective data outcomes) in line with the phenomenon of transcription effectiveness.

### Time saving

Jiegnote posed a series of superior value propositions compared to the traditional transcription methods. Participants felt that the software was better than the old (traditional) methods in implementing the transcription processes for it saved time. Rather than taking 6 h to transcribe one-hours audio recording, one can transcribe two audios instead.

*“I found this software very useful because it is very time saving. It is very time saving because with the traditional methods, we spend a lot of time, pausing and playing and going back and forth, so, to have a software that does that for you as you do what you supposed to do, that is the typing is very good, yes, very useful.”* Participant 5.

*“Jiegnote is proving to be easier and faster and so that’s how it is solving problems. The benefit of the software is speed for in case you are taking two days to finish a piece of work, maybe it will take you one day. The software eases work but it is majorly speedy.”* Participant 2.

### Improves the quality of transcripts

Participants reported that, on using Jiegnote, the quality of transcripts was improved compared to the traditional methods. Through its functions, the Jiegnote software limited word omission errors due to the looping technology (algorithm) that makes recorded audios clearer on listening.

*“The software is clearer in audio output compared to traditional, even if someone…., even if the interviewee is so fast, you can easily get the actual words. I liked the looping technology for it can keep playing repetitively at the same time you are typing and you don’t miss out words from the audio while using it.” it keeps playing, yah, I would say it improves the quality of the interview.*” Participant 7.

*“Jiegnote solves that part of missing out on some information that the interviewer or the respondent says. With Jiegnote, at least you cannot miss out on anything. The loop helps you not to miss the information.”* Participant 25.

We further provided questionnaires to participants, to rate the software in terms of effectiveness in the optimization of the qualitative research transcription process.

In both group 1 and 2, majority of the participants evaluated Jiegnote to be effective in the optimization of the transcription process in qualitative research at a rate of 8 out of 10 (Table 6).

**Table 6:** Effectiveness of the Jiegnote transcription software

Scale	Participant responses (frequency)		Total
	Group 1	Group 2	
10	1	0	1 (4.54)
9	2	3	5 (22.73)
8	3	7	10 (45.45)
7	2	1	3 (13.64)
6	1	0	1 (4.54)
5	0	1	1 (4.54)
4	1	0	1 (4.54)
3			
2			
1			
Total	10 (45.45%)	12 (54.55%)	22 (100%)

## Discussion

The manual transcription methods that require researchers to transcribe audio files using two software programs are error prone and time consuming. Through intrapreneurship and technological innovation, the study led to the development of a newer transcription software, whose functions (algorithm) led to a noticeable impact in improving the quality of transcripts and total time taken to transcribe audio files during qualitative research processes.

There are a few academic papers focused on the provision of guidance to employees and managers on how to implement intrapreneurship, and that few managers are able to articulate and communicate the process of intrapreneurship to their subordinates (Desouza, 2011), therefore, the study pragmatically provides an academic reference, and a practical demonstration on how intrapreneurship can be implemented and used to improve a value chain in an organizational setting.

The arithmetic average completion time was lesser when participants used the Jiegnote software compared to the traditional methods. Furthermore, on considering the effect size (Hedges' g), there was a noticeable impact the Jiegnote software had on the transcription process. This is due to the convenient, optimized and reengineered transcription work approach that Jiegnote offered. With Jiegnote, participants worked with one interface which rendered their work process faster compared to the traditional methods that involve two software applications to accomplish tasks.

The Jiegnote software generally yielded fewer transcription errors compared to the traditional methods, and on considering the effect size (Hedge’s *g*), the impact it had on the quality of transcript was small for the software was a working prototype at the time of the study. It lacked auto spelling correction functions, and therefore, it was prone to having a small effect on the total number of errors committed during transcription.

According to Holstein and Gubrium (2003), discrepancies between what is transcribed and the actual interview recording were noticed in some qualitative research study initiatives, and that, researchers hardly gave transcription quality a priority. The data outcomes on the factor of speed versus magnitude of errors (quality) implicated that, all participants who took the minimum time to complete the transcription of their respective audio cases, committed more total transcription errors, and so yielded poorer quality of transcripts compared to their counterparts who took the maximum transcription time on similar cases regardless of either using Jiegnote or the traditional methods, and therefore, there was an inverse relationship between speed and quality of transcript per audio case completed by a transcriber. This variance can be associated with Holstein and Gubrium findings whereby, some participants did not give sufficient attention to the quality of transcripts they were yielding.

Hennink and Weber (2013) implicated that, transcription was time and labor intensive, and that, it was often implemented by a variety of professionals like administrative staff, graduate students or non-research professionals with varying skills, training and supervision which posed a risk of poorly transcribed data. The researchers noted that the use of multiple transcribers led to variance in the quality and content of the transcribed data. The Jiegnote software study had respondents with varying professions, native languages and work experiences, this led to variance in the quality of the transcripts yielded regardless of the transcription method used.

The Jiegnote media looping algorithm fostered clearer audio outputs and information reemphasis because, parts of an audio file were repetitively played over a period of time until the next loop (Jump) functions were enabled to shift the repetitions to another section of the audio file. This led to fewer transcription errors upon using Jiegnote.

### Limitations

The Covid-19 pandemic prevented some participants from travelling to the study sites as there was limited access to public transport and yet some individuals lived distant places. They felt it inconveniencing to attend all the three visit schedules (Pre-intervention, intervention and post intervention) that were on different days and weeks. Some participants were employees and hence were actively involved in their designated work duties, consequently, some study activities were not completed and hence some data not analyzed, for example, participants who transcribed two audio cases instead of four, led to data exclusion during analysis.

According to the feedback attained from the participants, the audio cases used in the study were very long for a transcription process in such a research project, and hence, some did not complete the transcription task for the four audio cases. The participants were advised to complete the transcription tasks from their convenient environments like homes. This led to the lack of supervision pertaining to their recordings on the time taken to transcribe an audio file, hence posing a risk of inaccuracy in the recorded transcription completion time. The data outcomes on the participants who took the maximum and minimum transcription time (Table 5), showed that in both group 1 and 2, some participants were constant at taking the least, and most time in completing the transcription of audio cases. Therefore, this may implicate consistency in the accurate recording of the total time taken to complete the transcription of an audio file.

## Conclusion

The study demonstrates utilities associated with intrapreneurship and technological innovation in an organization setting whereby, the Jiegnote technology that was developed by the researchers, had some impact on the optimization of the qualitative research value chain. This was observed through the effect size (impact) evaluations conducted to investigate the superiority of the Jiegnote software against the traditional transcription methods in minimizing the average number of errors committed, and time taken to complete a transcription process.

### Recommendations

The Jiegnote software study implicates that, there is need to provide guidance to employees and managers on how to implement intrapreneurship and that, institutions should invest resources in intrapreneurship for it fosters; new product line and services development, employee creativity and engagement, knowledge creation, and innovation, and as a result, competitive advantage, new revenue streams, and optimized resource management outcomes are achieved.

## Data Availability

All the data sets generated and analyzed in this study are included within the submission.

## Abbreviations

IRB: Institutional Review Board
TECH: Technology
UNCST: Uganda National Council for Science and Technology
IDI: Infectious Diseases Institute
RCT: Randomized Controlled Trial
